# The biological interplay between air pollutants and miRNAs regulation in cancer

**DOI:** 10.3389/fcell.2024.1343385

**Published:** 2024-02-16

**Authors:** Alessandro Giammona, Sofia Remedia, Danilo Porro, Alessia Lo Dico, Gloria Bertoli

**Affiliations:** ^1^ Institute of Molecular Bioimaging and Physiology (IBFM), National Research Council (CNR), Segrate, Italy; ^2^ National Biodiversity Future Center (NBFC), Palermo, Italy; ^3^ Department of Pathophysiology and Transplantation, Università degli Studi di Milano, Segrate, Italy

**Keywords:** PM2.5, microRNAs (miRNAs), particulate matter, pollution, lung

## Abstract

Air pollution, especially fine particulate matter (PM2.5, with an aerodynamic diameter of less than 2.5 μm), represents a risk factor for human health. Many studies, regarding cancer onset and progression, correlated with the short and/or long exposition to PM2.5. This is mainly mediated by the ability of PM2.5 to reach the pulmonary alveoli by penetrating into the blood circulation. This review recapitulates the methodologies used to study PM2.5 in cellular models and the downstream effects on the main molecular pathways implicated in cancer. We report a set of data from the literature, that describe the involvement of miRNAs or long noncoding RNAs on the main biological processes involved in oxidative stress, inflammation, autophagy (PI3K), cell proliferation (NFkB, STAT3), and EMT (Notch, AKT, Wnt/β-catenin) pathways. microRNAs, as well as gene expression profile, responds to air pollution environment modulating some key genes involved in epigenetic modification or in key mediators of the biological processes described below. In this review, we provide some scientific evidences about the thigh correlation between miRNAs dysregulation, PM2.5 exposition, and gene pathways involved in cancer progression.

## Introduction

As reported by the World Health Organization in 2016, over 4.2 million deaths are caused by ambient air pollution, which is considered a worldwide public health emergency. In 2019, 99% of the world’s habitats live in places where air quality exceeds WHO limits and where the updated WHO Air Quality Guidelines (AQG) level for annual average PM concentration was not met (WHO, Ambient Air Pollution, 2021; Faridi et al., 2023).

Air pollution is defined as a combination of several components such as gases, particles, and several biological factors. In particular, particles cover a prominent role in the pathogenic role (Thangavel et al., 2022).

Particulate matter (PM), also called particle pollution, is a very complex and heterogeneous mixture of solid particles as metals, salts, organic chemicals generally adsorbed on carbonaceous core, and biological materials found in the air (Dergham et al., 2012; Burkholder et al., 2017). The International Agency for Research on Cancer (IARC) included the different PM classes within carcinogen categories and many epidemiologic studies collected in Asia, Europe, and North America highlighted a strong positive association between lung cancer and PM exposure and other air pollution components. Even though reports demonstrated a clear link between PM and cancers, there is still lack a of clear insight into the specific molecular mechanisms involved in cancer arising (Hoek and Raaschou-Nielsen, 2014; Loomis et al., 2014; Turner et al., 2014).

As a definition, PM is classified based on its aerodynamic diameter size in PM10 (particles with a diameter ≤10 µm) defined coarse ambient particulates; PM2.5 (particles with diameters ≤2.5 µm) indicated as fine particulate, and submicrometric PM0.1 (particles with a diameter ≤0.1 µm). The ambient fine particulate matter PM2.5 is one of the most prevalent components of atmospheric air particulate. It is composed of particles released by the natural sources of exhausted emission from fossil fuel combustion, normally emitted by cars, industry, or cigarette smoke, and biomass, by transition metals, organic compounds, biologic compounds, and acidic secondary pollutants (nitrate and sulfate); (Burkholder et al., 2017; Beelen et al., 2015; Shiraiwa et al., 2017; Iskandar et al., 2019; Turner et al., 2014). In addition, chemical reactions between precursor gases represent a furthermore hazardous air pollution elements defined as a secondary emission source (Zhou et al., 2021; Lara et al., 2023).

This review is focused on the effect of PM2.5 exposure on human health, cause this one is much worse compared to that of PM10 and investigate the effect of PM2.5 with acute or long-term exposure to humans and mice. The higher seriousness of PM2.5 is due to their higher amount and long distances in atmospheric air but also because of their smaller size, which allows them to reach alveolar structures in the lung. Therefore, the PM2.5 once inhaled into the lungs, could pass through the blood-air barrier into the blood circulation and then reach other tissues and organs, damaging the respiratory, central nervous system, and reproductive systems (Zhang et al., 2016; Cai et al., 2019; Wang et al., 2020).

Hence PM2.5 represents a severe risk factor for human health correlating with high hospitalization rate and mortality. The global burden of disease (GBD) study estimated that PM2.5 caused nearly 5 million deaths globally in 2017 (Pope et al., 2002; Xing et al., 2016; Wang et al., 2021).

Epidemiological studies have highlighted the impact of PM2.5 on human cardiovascular diseases and toxicological studies showed that it can induce several pathological effects such as cytotoxicity, oxidative stress, DNA damage, mutagenesis, immune and inflammatory responses. (Valavanidis et al., 2013; Szigeti et al., 2016; Szigeti et al., 2016; Pun et al., 2017). Scientific evidence showed that animal model PM2.5 exposure leads to lung damage, cardiac cell apoptosis, and right ventricle hypertrophy (Gao et al., 2020; Ding H et al., 2021).

Although there is much evidence indicating that exposure to air pollution PM affects human health, there is still not a clear understanding of all biological and molecular mechanisms regulating the detrimental role of PM 2.5. What is known is that heart and lung dialogue involve three main aspects: gas exchange, body fluids, and mechanical interaction, however, there is no evidence that demonstrates a direct PM2.5 deposit into blood vessels. Moreover, many scientific groups have shed light on different molecules involved in the promotion of cardiac disease or lung cancer.

MicroRNAs (miRNAs) are a class of endogenous, non-coding single-stranded small RNAs composed of 20–24 nucleotides. It has been widely demonstrated that miRNAs play a key role in the regulation of protein translation and transcription. Mechanistically, miRNAs bind the complementary sequence within the 3′-untranslated region of target messenger RNA blocking the translation of a variety of target proteins. Functionally, miRNAs regulate several cellular processes involved in cell proliferation, differentiation, apoptosis, and metabolism (Hwang and Mendell, 2006). In oncology, for instance, miRNAs are able to regulate oncogenes and oncosuppressors sustaining or inhibiting cancer onset and progression (Otmani and Lewalle, 2021).

Scientists have begun to investigate the connection between miRNA deregulation, air pollution in terms of environmental exposure, and the risk of disease development, including cancers (Mancini et al., 2020; Feng et al., 2019). Moreover, another class of RNA molecules was associated with air pollution, the long non-coding RNAs (lncRNAs), which are longer than 200 nucleotides with limited protein-coding ability and that was already associated with Epithelial-Mesenchymal Transition (EMT) and human cancers. For instance, lncRNAs can guide the Heat shock proteins (HSPs) that are involved in different kinds of stress, including those activated after exposure to air pollution stressors (Shamovsky et al., 2006) also to genotoxic agents, ultraviolet (UV)-C irradiation, oxidative stress, cigarette smoke extracts, heavy metals, and endocrine disrupting chemicals (Turner et al., 2014).

### The methodological approaches for the *in vitro* assay exposure to PM 2.5

One aim of our review is to collect and describe the main *in vitro* approaches used by researchers to investigate the exposure to PM 2.5 effect using *in vitro* models. The exact mechanism associated with the impact of PM2.5 on human physiology is still unclear. One hypothesis supposes that inhaled PM2.5 accumulates on the lung epithelium, activating inflammation, releasing inflammatory mediators and stimulating alveolar receptors, which in turn activate the autonomous nervous system and the neuroendocrine pathway (Wu et al., 2016). Another possibility regards the translocation of PM via the pulmonary epithelium to vascular circulation, affecting all the tissues, and causing systemic pathologies (Bai et al., 2018).

For this reason, the lung cells represent the gold standard model, as documented in the literature: indeed, the adenocarcinoma human alveolar basal epithelial cells (A549) or the SV40 large T antigen immortalized human bronchial epithelial cell line (BEAS-2B) are the most used and characterized cell models, when it is not available a patient cohort of NSCLC. Those cells are cultured in 2-dimension monolayer, maintained with keratinocyte basal medium (KBM) and supplemented with human epidermal growth factor, insulin, hydrocortisone, calcium, bovine pituitary extract, gentamicin sulfate, amphotericin-B (GA-1000) (Fuentes-Mattei et al., 2010; Dergham et al., 2012). The cell models are usually subjected to two different time exposure to PM: the acute short-term and the chronic long-term one. The acute exposure, as defined by the literature, is the exposition to a range of PM 2.5 from 0 to 200 μg/mL for 24hs, or the exposition to PM 2.5 from 0 to 100 μg/mL each 24/48 h for a total of 5 cell passages (Kloog et al., 2013). On the contrary, increased exposure time points than the ones previously described generally were identified as chronic exposure.

Technically, it has been ascertained by the scientific community that the standardized method, that it is also the more efficient in collection of the PM2.5 is based on the exposition to the cells to PM2.5 on Teflon-based filter (Chen et al., 2020). Then, collected in this way, are usually resuspended in water or in organic solvent before the characterization of the morphology, size, concentration and phenotype of particles, through transmission electron microscopy. The suspension is then melted in the cell medium for the following biochemical or biological experimental settings. The main limit of this methodology is mainly that the particulate matter is not fully representative of what is happening the atmosphere, among all for the humidity that with this method does not recapitulate the real atmosphere humidity features. Other limits of this methodology are due to the different sampling collection time, the extraction method and the administration action of PM2.5, reported in the literature. In particular, as shown in the Fuentes-Mattei paper, the authors set the airflow rate at 17 L/min and each filter collected the material long 72h, and the PM2.5 was extracted by Soxhlet, based on the EPA method 3540C (U.S. EPA, 1996). The organic PM2.5 was sequentially extracted using hexane as an organic solvent for the non-polar fraction, followed by acetone to obtain the polar organic fractions. The recovered solvent was dried by means of rotor evaporation to approximately 1–2 mL and subsequently under a gentle stream of nitrogen. The vials with organic extracts were weighed and the content dissolved in dimethyl sulfoxide (DMSO) following the published method described by the Molinelli group (Fuentes-Mattei et al., 2010). Instead, the Wang group collected the PM2.5 samples from a longer time spot from 26th November to 21 December 2014 with large-volume PM2.5 samplers (Intelligent 2031; Qingdao Laoying Inc., China). The authors used an ultrasonic extraction technique to obtain the PM2.5 suspensions placing the sampling filters into a sterilized beaker containing cool sterilized ultrapure water and sonicated for 30 min. Therefore, to obtain the PM2.5 suspensions the extracted liquids were filtered with sterilized gauze. The suspensions were freeze-dried under vacuum (Labconco, Kansas, USA) to obtain the PM2.5 particles. The prepared samples were weighed and stored at −20 °C (Wei et al., 2017).

In the Xie Chao paper, they described a procedure for PM2.5 sampling using a quartz filter (8 cm × 10 cm, 2500QAT-UP, Pallflex Products, Putnam, USA) collecting continuously or weekly in the central area of Beijing (China) from January 2017 to June 2017 at a flow rate of 180 L/min. The sampling was treated with anhydrous alcohol and dissolved in pyrogen-free water. Subsequently, the extraction was sonicated for 2 days in an ultrasonic box and then concentrated through vacuum freeze-drying. Next, the double-distilled water was added to the freeze-dried product, followed by centrifugation at 5,000 rpm (Chao et al., 2020).

At the end of the extraction process, the PM 2,5 particles were resuspended in the culture medium. However, even though this kind of treatment may simulate cell exposure to PM, it is not completely the same as what the *in vivo* epithelial respiratory cells undergo in polluted areas.

Of note, even though we found papers that describe the difference between the two methods, both methods are involved in the stimulation of the malignant phenotype (Sima et al., 2021). However, the acute PM2.5 exposition is more involved in the upregulation of EMT markers without significant changes in cell morphology or in stem cells (Wei et al., 2017). Instead, chronic exposure increases not only the genes involved in the EMT process as mRNAs as well as proteins but exerts its effect also on the expression of a critical pool of microRNAs involved in the cancer stem cells (CSC) features and tumorigenesis (Wang et al., 2020).

In fact, as observed by Huang et al. (2003), the BEAS-2B cells exposed for 8h to 100 μg/mL of PM2.5 particles did not show a significant alteration of viability compared to untreated cells, but the fine and ultrafine particles would be responsible for inflammatory cytokines induction, possibly due to their metal content (Mn and Cr) On the contrary, PM 2.5 exerts its effect on cell cytotoxicity, with a significant cell reduction after increasing doses of PM2.5. Therefore, the authors concluded that the harmful effect of PM2.5 exposure highly depends on the dose and not only on time. (Huang et al., 2003).

Consequently, a crucial point of these kinds of studies is the amount of PM2-5 that can reach the surface of the cells is limited and not easily quantified. As described by Wei H’s study the filter used for the preparation can hold the same particles. Finally, the step-in water can change the starting size and the grade of humidity in the preparation (Wei et al., 2017). Recently, an innovative methodology proposed the use of air-liquid interface (ALI) culture method (Ghio et al., 2013) to allow the direct exposure of the lung epithelial cells to atmospheric air pollution (Costabile et al., 2023). In this paper, the molecular analysis revealed the activation of an anti-oxidant defense, mediated by Heme Oxygenase expression, and the activation of pro-inflammatory chemokine ligand CXCL8 in lung normal epithelial cells.

## Biological effects and impact on miRNAs

PM2.5 exposure is responsible for severe diseases in the respiratory and cardiovascular systems (Valavanidis et al., 2013; Szigeti et al., 2016).

This review aims to describe the link between the different biological effects and PM.,5 exposure.

Among the most important cell stress effects due to PM2.5 exposure, we have mentioned the cycle and proliferation change, big oxidative stress due to the redox system modifications, apoptotic machinery deregulation, inflammation induction, and promotion of Epithelial-mesenchymal transition (EMT). Even if, this subclassifying revealed that several of these topics overlap.

Moreover, our review focus is to document how PM2.5 exposure acts both on down or upregulation of many miRNAs, whose activity is responsible for the control of tumour suppressor or tumour-promoting genes.

Therefore, thanks to this studio on miRNA deregulation by PM2.5 exposure we would like to stress the importance of this molecular mechanism investigation, to discover a reliable pathologies’ prognosis mechanism or biomarker, in particular related to cancer progression, and to identify a reliable therapeutic molecular target.

Many groups investigated its contribution to the changes in miRNA expression due to PMs in airway epithelial cells. As mentioned, many miRNAs have been already described to have a role in the control of lung cancer and an association between diesel exhaust and lung cancer has been reported by the Environmental Protection Agency (EPA) in 2002 (Weir, 2002). Nevertheless, the molecular mechanisms by which the DEP initiates disease are still elusive.

In the Jardim et al. (2009), a massive miRNA profile was performed by Agilent microarray, where human bronchial epithelial cells were grown in ALI condition, exposed for 2h to 10µg/cm2 of resuspended diesel exhaust particles (Jardim et al., 2009), Seven hundreds twenty-three miRNAs were detected and analyzed: 130 miRNAs showed an increase of ≥1.5-fold in response to DEP, whereas 67 showed a decrease of ≥1.5-fold in expression.

Therefore, as shown in the below Table 1, the mRNAs induced or repressed ≥ 4-fold in cells exposed to 10 μg/cm2 DEP are: miR-513b/c, miR-513a-5p, miR-923, miR-494, miR-338-5p (all upregulated) and miR-31, miR-26b, miR-96, miR-27a, miR-135b, miR-374 (all downregulated).

**TABLE 1:** list of up- (left) or downregulated miRNAs obtained in microarray analysis on human bronchial cells exposed to PM2.5 2 h in ALI condition.

Upregulated	Downregulated
miR-513c	miR-31
miR-513b	miR-26b
miR-513a-5p	miR-96
miR-923	miR-27a
miR-494	miR-135b
miR-338-5p	and miR-374a

The analysis of the putative network of targets regulated by these miRNAs revealed that:

miR-513a-5p, miR-494, miR-96 are able to control inflammatory response, by targeting IL8, NFkB, and CXCR4.

Diesel exhaust particles (DEPs) the miR-21 expression in HBE cells, A549, and treated HBE cells were profiled. The analysis demonstrated that miR-21 is overexpressed by greater than 16-fold in untreated A549 cells compared with the untreated HBE cells. The miR-21 expression in the HBE cells treated with DEPs for 48 h was 6-fold higher than the untreated HBE cells.

The effect of DEPs on PTEN/PI3K/AKT activity was then measured (Washam, 2009).

### Molecular pathway associated to cell proliferation and survival

Mei Yang et al. have shown that 24 h of PM2.5 exposure in lung A549 cancer cells induces cell proliferation and enhances migration and invasion. The authors demonstrated that PM2.5 exposure is directly responsible for miR-582-3p upregulation. Indeed, when they silenced that miRNA, observed a significant reduction of cancer aggressiveness in terms of proliferation, migration, and invasiveness related to the positive modulation of the Wnt/β-catenin signalling by miR-582-3p. However, this study lacks the analysis of the expression level of Wnt/β-catenin target genes and *in vivo* studies will be necessary to validate *in vitro* data (Yang et al., 2022).

In another paper, three miRNAs were selected a signature for oncogenesis in human bronchial epithelial (HBE) cells by acting on c-fos gene (Cai et al., 2021). In detail, the authors showed an upregulation for miR-25−3p and miR-215−5p and a downregulation for miR145-5p after PM2.5 exposure at the concentration of 50 μg/mL for 24 h. MiR-25, is a member of the miR-106b-25 cluster and it has been reported playing an important role in many biological processes as an oncogenic MicroRNA. MiR-25-3p is upregulated in triple-negative breast cancer (TNBC), where it promotes *in vitro* cell proliferation and tumor growth in a xenograft model and inhibits apoptosis through the Hippo pathway in Osteosarcoma (Chen et al., 2020; Rao et al., 2020).

Instead, the miR-215-5p is considered a master regulator of autophagy, by targeting PI3K and activating ROS-mediated MAPK pathways in the cardiomyocytes (Cai J et al., 2021; Xiong et al., 2021) and in the Non-small Cell Lung Cancer (NSCLC; Fan et al., 2021; Niranjan et al., 2021).

It was documented that the miR145-5p in NSCLC cells is implicated in EMT in acting on the c-Jun N-terminal kinase (JNK) signaling pathway (Chang et al., 2017).

The effects of PM2.5 stimulation on cell cycle and proliferation are also related to NF-κB activation. The authors observed that the PM2.5 exposure (with a concentration range from 100 to 500 μg/mL depending on assay typology) is able to induce the miR-155 expression, through an increase of NF-κB level. What they observed was that miR-155 promotes STAT3 signaling pathway activation and a downregulation of SOCS1 level inducing uncontrolled cell proliferation in the HBE model (Xiao et al., 2019).

### Epithelial-mesenchymal transition (EMT)

Wang Y. observed that PM2.5 exposure by 50 and 100 μg/mL downregulated miR-139-5p. As shown by the authors the decrease of miR-139-5p promotes an upregulation of Notch protein causing the EMT. Instead, the single increase of miR139-5p is able to revert the EMT transition (Wang et al., 2020).

Instead, Luo F. highlights the link between PM2.5 stimulation, lung injury and miRNA expression. In this work, the long non-coding MALAT1 (lnc-MALAT1) is associated with PM2.5 chronic exposure in lung bronchial epithelial cells (HBE), which regulated the expression of the EMT-associated genes such as ZEB1, ZEB2, Slug, and Snai1 (Luo et al., 2019).

The authors showed that the exposition for ∼15 weeks, over 30 passages to PM2.5 at the concentration of 5 μg/mL is able to modify the bronchial epithelial cells’ anchorage ability and their morphology. The authors indicated that the molecular loop after PM 2.5 exposition is the increased expression of the NF-κB signaling pathway that promotes MALAT-1 induction. This last interacts with miR-204, impairing its expression and preventing the molecular inhibition of ZEB 1 expression. In the end, this miRNA abrogation causes the increase of the EMT and the transition to the most aggressive profile of the lung cancer cells. Also, in this case, the chronic PM2.5 exposition supports the oncogenic changes that support malignant transformation in the lung (Luo et al., 2019).

Other evidence between EMT transition and PM2.5 exposure has been recently provided and it was explored the difference between acute and chronic exposure. The chronic treatment of lung cancer cells revealed a stronger effect. Indeed, although acute exposure supports the malignant transformation of the cells it remains bordering with the EMT factors induction without a big effect on cell morphology or stem potentiality. Instead, chronic exposure, such as 5 μg/mL for 24/48 h for five passages, in A549 cells it was showed a malignant transformation through the induction of EMT markers and the acquiring of a mesenchymal morphology. Moreover, cells increased the expression of the stem markers SOX2 and OCT4 (Fu et al., 2021).

The authors showed that chronic exposure to PM2.5 abolished the expression of a miRNAs pool that is classified as a negative EMT regulator. In particular, they found downregulated: let-7a (a negative regulator of EMT), miR-15, miR-16 (a negative regulator of cell cycle), and miR-34a (that regulates SNAIL; Mathieu and Ruohola-Baker, 2013; Wei et al., 2017).

In another paper, the authors used a microarray technology from whole blood samples exposure to PM2.5 to identify seven microRNAs (miR-24-3p, miR-4454, miR-4763-3p, miR-425-5p, let-7d-5p, miR-502-5p, miR-505-3p) (Mancini et al., 2020).

One of the most deregulated miRNAs in the context of air pollution exposure is miR-222, which appears to be deregulated in many cancers including NSCLC. Among miRNA targets, there is the tumor suppressor PTEN (TIMP3) causing enhanced cellular migration by the activation of the AKT pathway.

The expression was also associated not only with PM2.5 exposure but to mixtures of air pollutants, specifically PM10, PM2.5, UFP, black carbon, organic carbon, sulfates, and ozone (Quezada-Maldonado et al., 2018; Sima et al., 2021).

### Inflammatory response

It has been demonstrated that PM2.5 is also involved in the onset and maintenance of inflammatory response. Zhao et al. described that in HBE cells PM2.5 exposure induced miR-9-5p expression and this modulating the NF-κB signaling pathway (Zhao et al., 2020) In particular, authors demonstrated that lncRNA RP11-86H7.1 was induced after PM2.5 stimulation and was involved in the inflammatory response.

MiR-9-5p activity impaires IL-6, IL-8, TNF-α, p-IκB-α, p-p65, and Iκκβ expression. Concomitantly, its upregulation blocked also NF-κB expression.

Li X. et al. enforced the lncRNA role in the inflammatory response. The authors showed that PM2.5 exposure, 75 μg/mL for 24 h induced IL-6 and IL-8 increase in the BEAS-2B cells.

Authors argued that a key role on that was covered by circRNA104250 and lncRNAuc001 (Li et al., 2020).

Moreover, other evidence suggested that another long non coding RNA, the lnc-PCK1-2:1, promotes the IL-6 and IL-8 secretion after exposure to PM2.5 through the miR-3607-5p and NF-κB signaling pathways (Pan et al., 2019).

### Oxidative stress

A recent publication demonstrated in asthma that the PM2.5 exposition induce a massive ROS production promoting a strong inflammatory response. It was observed 12 miRNAs associated with different level of asthma severity. The main responsible for the ROS release it was discovered in miR-206, which abrogates SOD and NRF2 antioxidant factors in primary mouse tracheal epithelial cells. In details miR-206, induced by PM2.5. targets the 3′UTR of SOD inhibiting its expression and its anti-oxidant defense, promoting ROS accumulation (Wang et al., 2019).

As mentioned before, Zhang F. described miRNAs induced after PM exposure and in particular the miR-25-3p promoted a metabolism changes, such as lactate modulation, triglyceride (TG) adenosine triphosphate (ATP) content, but inducing also a big release of reactive oxygen species (ROS) (Zhang et al., 2016).

Other two papers by Jeong SC. and Liu C. described the consequence of water (W-PM2.5) or organic (O-PM2.5) PM2.5 exposure in A549 cells; the cells were exposed either to water (W-PM2.5) or to organic (O-PM2.5) soluble extract for 48 h. A total of 37 miRNAs and 62 miRNAs were altered 1.3-fold in W-PM2.5 and O-PM2.5, respectively. W-PM2.5 altered genes are involved in responses to nutrients, positive regulation of biosynthetic processes, positive regulation of nucleobase, nucleoside, and nucleotide, and nucleic acid metabolic processes; conversely, the 69 O-PM2.5 altered genes are involved in DNA replication, cell cycle processes, the M phase, and the cell cycle checkpoint (Liu et al., 2015; Jeong et al., 2017; Veerappan et al., 2019).

**FIGURE 1:**
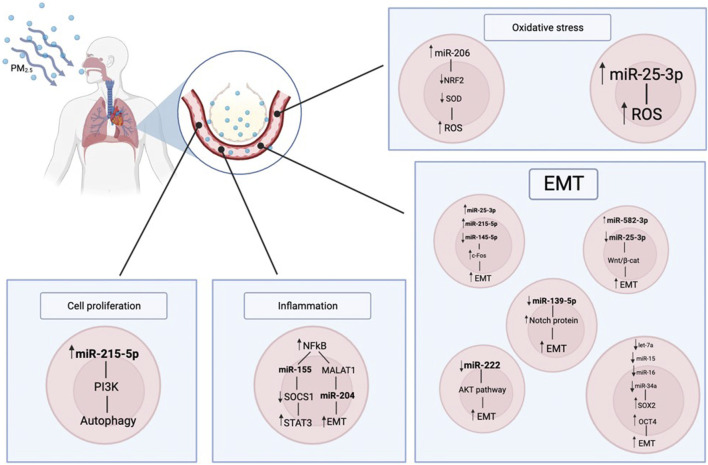
Here, it is represented how the PM2.5 can impact on the human body. The air particulate is inhaled, reaches the pulmonary alveoli and it is transferred to the bloodstream; in this way it enters the cell metabolism, impairing it. In particular there are consequences on the miRNAs regulation, that in turns has effects on the inflammation state, cell proliferation and oxidative stress.

**FIGURE 2:**
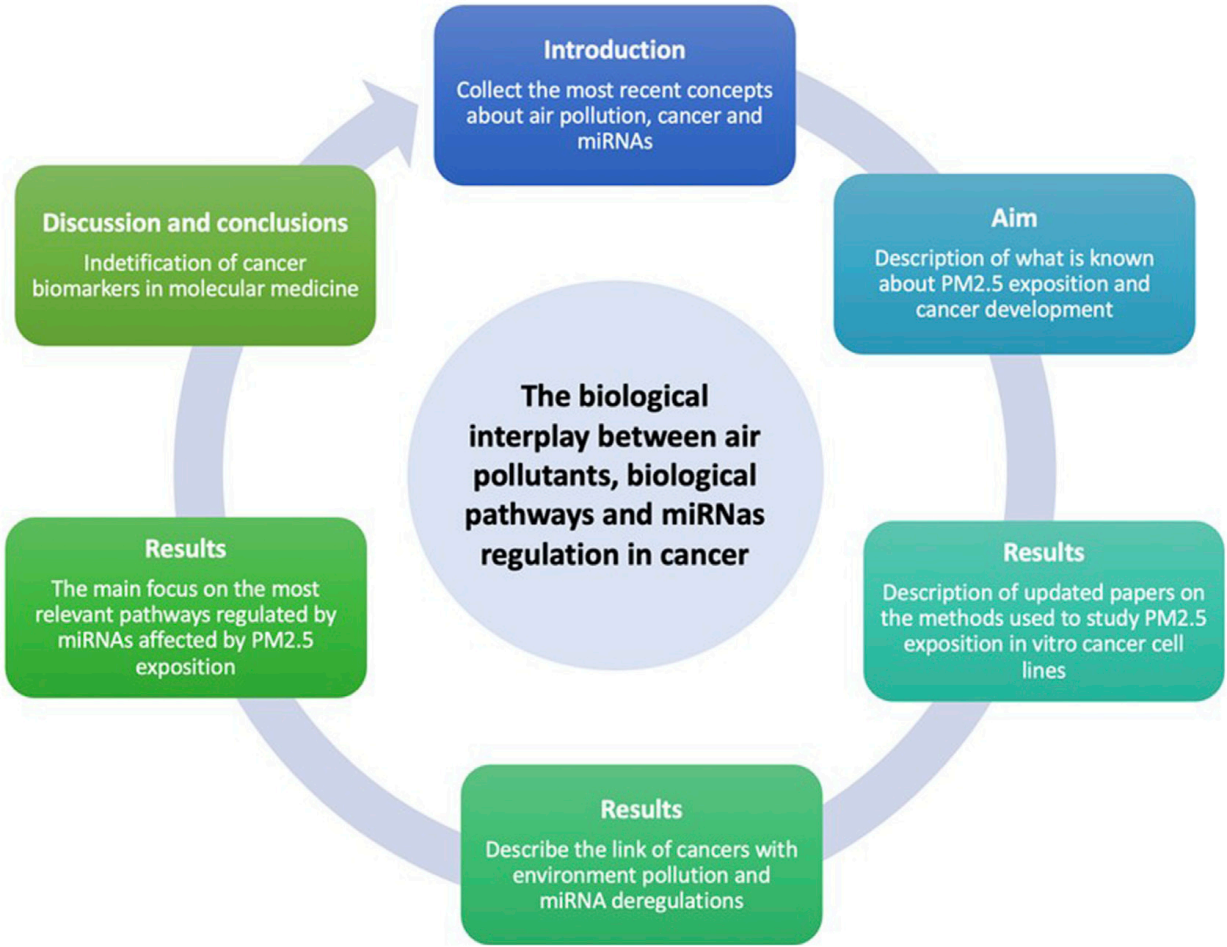
In this review it will explore the PM2.5 effects on cells in particular for what concerns the release of miRNAs and the biological effects exerted by themselves. The proof of concepts is based on the research of the keywords miRNAs, air particulate, lung epithelial cell (20 oct 2022) on PubMed and bioRxiv and a selection of XX articles has been included in this review. The main focus of this review was the effects of PM2.5 mainly on the modulation of miRNAs in *in vitro* models, by comparing different methods of stimulation.

In this study, it was hypothesized that components of airborne PM2.5 may alter microRNA expression resulting in deregulation of their target oncogenes, which in turn may lead to carcinogenesis (as described in Table 1; Figure 1; Figure 2). To test this hypothesis, it was firstly examined the effects of PM2.5 on whole-genome microRNA expression in human bronchial epithelial (HBE) cells. Then, protein-coding genes were identified that may be targeted by PM2.5-modulated microRNAs (Zhou et al., 2015).

## Discussion and conclusion

**FIGURE 3:**
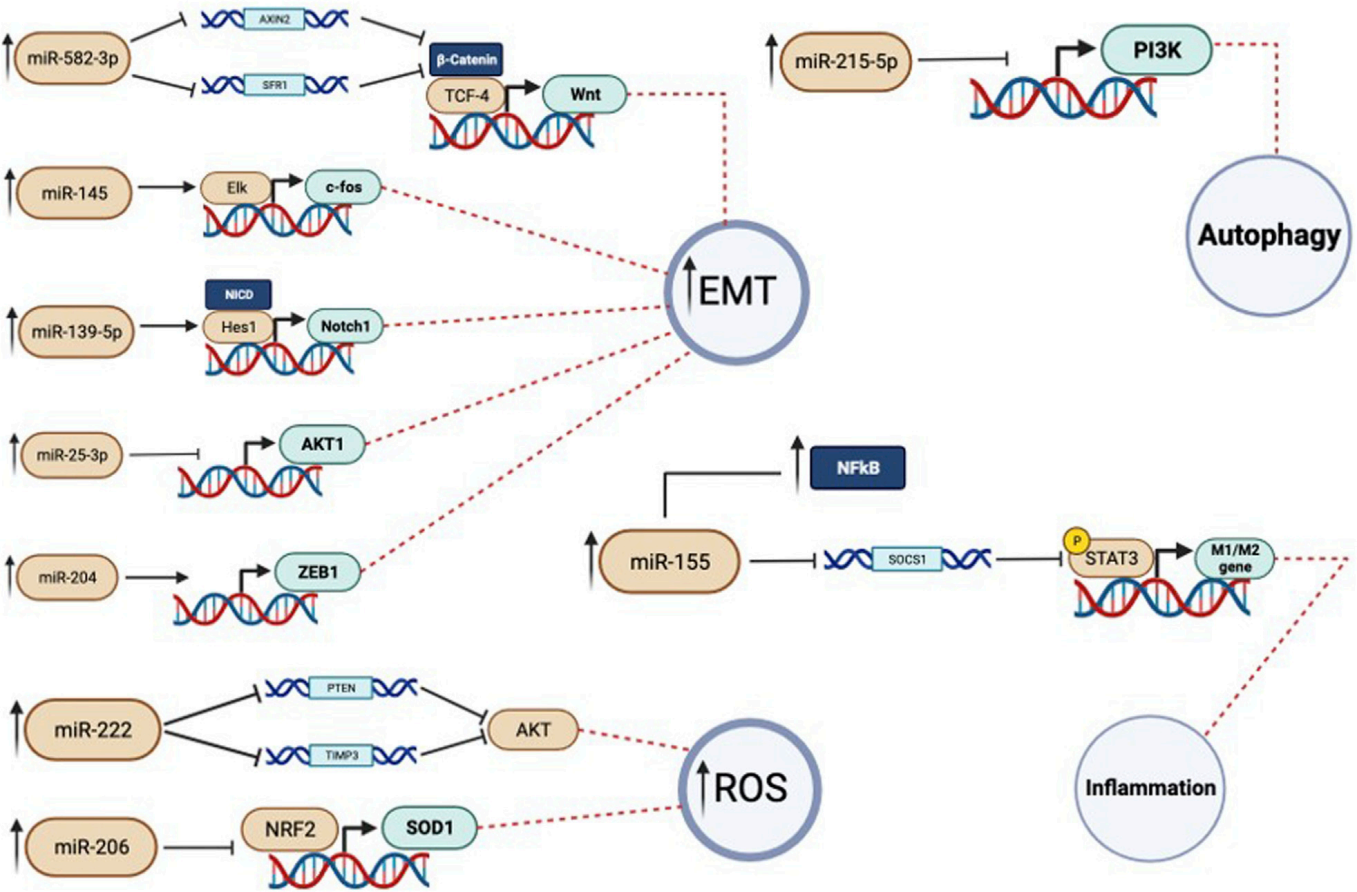
In this figure the miRNAs activity is showed more in detail. Each miRNA has a target gene on which operates. When there is the dysregulation, and so an overexpression, of a specific miRNA there is also an impairement in the expression of its target gene. In particular we can see that this can cause autophagy inhibition, inflammation, rise of oxidative stress and, in cancer cells, increase in the migration activity.

It is clear from the presented literature that air pollution exerts a critical effect on human health, in particular in the onset and progression of cancer diseases, where many evidences are reported on the main role of particulate matter in cancers. In this review, we have investigated at a molecular level which biological mechanisms correlated air pollution to cancers, focusing on the potential link between miRNA’s contribution and the PM 2.5 exposure. Although the central role of miRNAs in cancer biology, together with their survival mechanisms, is well known, there are few studies discussing the miRNA deregulation after PM 2.5 exposure. Beyond the different cell models used and the different methods of exposure to PM described in the literature, our analysis revealed that PM2,5 exposure causes a big re-arrangement of inflammatory and metabolic pathways and a concomitant increase of cell proliferation and invasiveness; interestingly, all of the above-cited readaptations, matched with and altered miRNA expression. In particular, miR-582-3p, miR-25-3p, miR-215-3p, miR-145-5p and miR-155, altered by PM exposure, were involved in cell proliferation and survival; miR-139-5p, miR-204, let-7a, miR-15, miR-16, miR-34a, miR-24-3p, miR-4454, miR-4763-3p, miR-425-5p, let-7d-5p, miR-502-5p, miR-505-3p are involved in EMT; miR-9-5p and miR-3607-5p were involved in inflammatory response and miR-206 and miR-25-3p control oxidative stress. Among the cancer cell pathways involved in this rearrangement, induced by PM 2.5 exposure, pathways related to NF-kB, inflammation and oxidative stress have been discussed in this review. Indeed, all the mentioned pathways are the main drivers of the tumor onset and progression, sustaining the biological processes involved in the higher invasiveness or apoptosis resistance (Figure 3; Figure 1). Obviously, other molecular pathways involved in the hyperproliferative phenotype of cancer cells could also be included in future studies in which the association between PM2.5 and cancer is closely interconnected.

In conclusion, considering that all the described responses shown by cancer cells to PM exposure involve miRNA deregulation, we speculate that these miRNAs could be considered as theranostic biomarkers since they allow to describe the biological effect in response to air pollution exposure evidencing specific target pathways, involved in cancer development, and meanwhile important in the cellular adaptation to particulate exposure. Thus, air pollution drives miRNA deregulation in cancer and these miRNAs could alter some key molecular pathways, involved in the most aggressive features of cancer.

The identification of key molecular pathways, modified by PM2.5 exposure, could be consider as an influencing factor in the development of a personalized medicine approach. Indeed, once the pathway has been identified, a therapeutic strategy aimed at targeting it could be set up. Finally, this review combines two fundamental needs for human health: understanding the effects of pollution on biological systems and identifying therapeutic options for cancer.
