# Supplementary material for: The biological interplay between air pollutants and miRNAs regulation in cancer
Source: Front Cell Dev Biol. 2024 Feb 16;12:1343385. doi: 10.3389/fcell.2024.1343385 (PMC10905188; doi:10.3389/fcell.2024.1343385)
Supplement: Supplementary file 1 [file Table1.DOCX]

| ***miRNA ID*** | ***Process*** | ***Type of particles*** | ***In vitro model*** | ***REF*** |
| --- | --- | --- | --- | --- |
| miR-582-3p **↑** | EMT process (Wnt/beta Cat signaling), cell proliferation and invasion processes | modulation of miRNA by PM2.5, 24h exposure | Lung cancer A549 cell line | 10.1007/s11356-021-16801-2 |
| miR-25-3p, -215-5p **↑**; miR-145-5p **↓** | c-Fos pathway | modulation of miRNA by PM2.5 (concentration), 24h - particles resuspended in water | Human bronchial epithelial cells (HBE) | <https://doi.org/10.1016/j.etap.2021.103607> |
| miR-25-3p **↑** | metabolism of lactate in breast cancer | modulation of miRNA by PM2.5 (concentration), 24h exposure | triple negative breast cancer | ([Chen et al., 2018](https://www.sciencedirect.com/science/article/pii/S1382668921000260#bib0030) |
| miR-215-5p | autophagy |  |  | ([Cai et al., 2019](https://www.sciencedirect.com/science/article/pii/S1382668921000260#bib0010)) |
| miR145-5p | JNK signaling pathway |  | Non-small cell lung cancer | ([Chang et al., 2017](https://www.sciencedirect.com/science/article/pii/S1382668921000260#bib0020)) |
| miR-139-5p ↓ | Notch pathway | PM2.5 (50 and 100ug/ml) | HBE | doi:10.7150/jca.46976 |
| miR-155 | NFkB signaling (STAT3 activation) resulting in cell proliferation | PM2.5 (2mg/ml) | HBE | NF-κB-regulation of miR-155, via SOCS1/STAT3, is involved in the PM2.5-accelerated cell cycle and proliferation of human bronchial epithelial cells) |
| miR-204  (Suppressed by H19 lncRNA and MALAt1) | EMT process (ZEB1, ZEB2, Slug, and Snai1) | PM2.5 (5 μg/m) | HBE | LncRNA MALAT1, an lncRNA acting via the miR-204/ZEB1 pathway, mediates the EMT induced by organic extract of PM2.5 in lung bronchial epithelial cells |
| let7a, miR-15, miR-16, miR-34a **↓** | EMT induction (increase in Sox2 and Oct4)(SNAIL) | PM2.5 (5 μg/mL for 24 ∼ 48 hours per passage, continued for five passages) | A549 lung cancer cell l ine | Mathieu J, Ruohola-Baker H. Regulation of stem cell populations by microRNAs. Adv Exp Med Biol. 2013;786:329–351 |
| Circulating miR-24-3p (↓); Circulating miR-4454, miR-4763-3p, miR-425-5p, let-7d-5p, miR-502-5p, miR-505-3p all **↑** | p53 signaling pathway, JAK- STAT signaling pathway, TGF-beta signaling pathway, Hippo signaling pathway | PM2.5 (24h exposure, <10ug/m3) | whole blood | doi: 10.1016/j.envpol.2020.114392. |
| miR-9-5p | NF-κB signaling pathway (IL-6, IL-8, TNF-α, p-IκB-α, p-p65, and Iκκβ expression) | PM2.5 (2 mg/ml) | human bronchial epithelial cells | LncRNA RP11-86H7.1 promotes airway inflammation induced by TRAPM2.5 by acting as a ceRNA of miRNA-9-5p to regulate NFKB1 in HBECS |
| miR-3607-5p regulated by circRNA104250 and lncRNAuc001.dgp.1 | NFkB pathway (IL-6, IL-8 induction) | PM2.5 (75 ug/ml) | BEAS-2A cell line | [Pan et al., 2019](https://www.sciencedirect.com/science/article/pii/S0269749119349437#bib30) |
| miR-206  (+ 12 miRNAs) | ROS production and release (SOD abrogation) | PM2.5 | primary mouse tracheal epithelial cells | PM2.5 inhibits SOD1 expression by up-regulating microRNA-206 and promotes ROS accumulation and disease progression in asthmatic mice |
| miR-513a-5p (up) | GNG13, GNAI2, GNG12, Rac, KRas, TRAF6 | IL8 signaling |  | doi: 10.1289/ehp.0900756 |
| miR-494 (up) | ROCK1, PROS1, MAPK, Ras, Ras homologue, Ap1, Rac, Akt, ERK, Pkc(s) | CXCR4 signaling |  | doi: 10.1289/ehp.0900756 |
| miR-96 (down) | KRAS SDC2, PI3K, Pkc(s), ERK, NFkB, p38 MAPK, AKT, MAPK, PRKCE;  PRMT5, SW1/SNF | NFkB signaling  control on methylation |  | doi: 10.1289/ehp.0900756  Pal S, Baiocchi RA, Byrd JC, Grever MR, Jacob ST, Sif S. 2007. Low levels of miR‑92b/96 induce PRMT5 translation and H3R8/H4R3 methylation in mantle cell lymphoma. EMBO J 26(15):3558–3569. |
| miR-513 | B7-H1 | INFgamma pathway and apoposis |  | Gong AY, Zhou R, Hu G, Li X, Splinter PL, O’Hara SP, et al. 2009. MicroRNA-513 regulates B7-H1 translation and is involved in IFN-gamma-induced B7-H1 expression in cholangiocytes. J Immunol 182(3):1325–1333. |

**Tab.1 List of miRNAs involved in cancer progression responding to PM2.5**
