# Supplementary material for: The biological interplay between air pollutants and miRNAs regulation in cancer
Source: Front Cell Dev Biol. 2024 Feb 16;12:1343385. doi: 10.3389/fcell.2024.1343385 (PMC10905188; doi:10.3389/fcell.2024.1343385)
Supplement: Supplementary file 2 [file Presentation1.PPTX]

## Slide 1
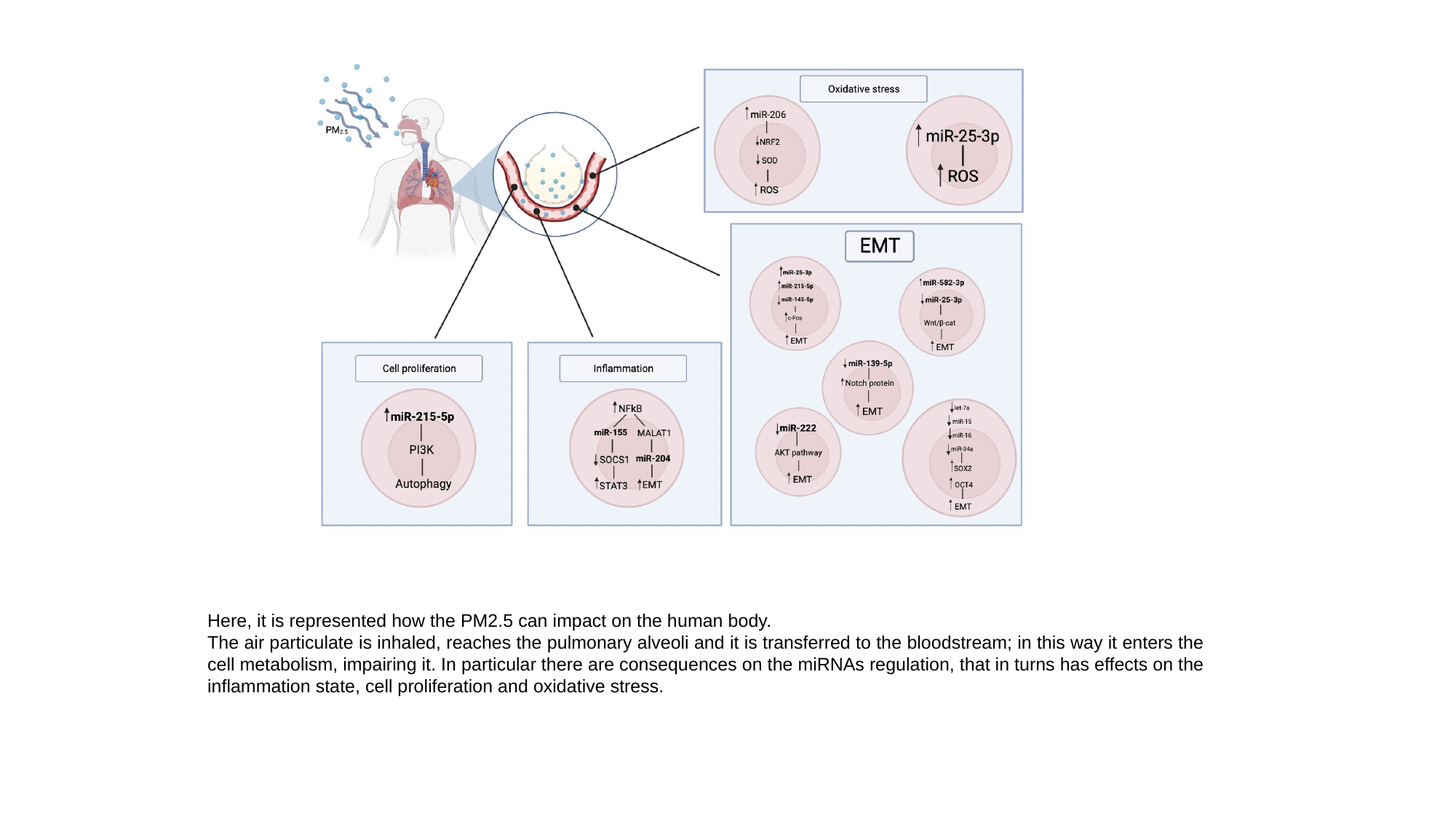

Here, it is represented how the PM2.5 can impact on the human body.
The air particulate is inhaled, reaches the pulmonary alveoli and it is transferred to the bloodstream; in this way it enters the cell metabolism, impairing it. In particular there are consequences on the miRNAs regulation, that in turns has effects on the inflammation state, cell proliferation and oxidative stress.
